# The Analysis of Gene Expression Data Incorporating Tumor Purity Information

**DOI:** 10.3389/fgene.2021.642759

**Published:** 2021-08-23

**Authors:** Seungjun Ahn, Tyler Grimes, Somnath Datta

**Affiliations:** Department of Biostatistics, University of Florida, Gainesville, FL, United States

**Keywords:** tumor purity, RNA-seq data, differential network analysis, differential gene expression analysis, gene expression data, confounding effects

## Abstract

The tumor microenvironment is composed of tumor cells, stroma cells, immune cells, blood vessels, and other associated non-cancerous cells. Gene expression measurements on tumor samples are an average over cells in the microenvironment. However, research questions often seek answers about tumor cells rather than the surrounding non-tumor tissue. Previous studies have suggested that the tumor purity (TP)—the proportion of tumor cells in a solid tumor sample—has a confounding effect on differential expression (DE) analysis of high vs. low survival groups. We investigate three ways incorporating the TP information in the two statistical methods used for analyzing gene expression data, namely, differential network (DN) analysis and DE analysis. Analysis 1 ignores the TP information completely, Analysis 2 uses a truncated sample by removing the low TP samples, and Analysis 3 uses TP as a covariate in the underlying statistical models. We use three gene expression data sets related to three different cancers from the Cancer Genome Atlas (TCGA) for our investigation. The networks from Analysis 2 have greater amount of differential connectivity in the two networks than that from Analysis 1 in all three cancer datasets. Similarly, Analysis 1 identified more differentially expressed genes than Analysis 2. Results of DN and DE analyses using Analysis 3 were mostly consistent with those of Analysis 1 across three cancers. However, Analysis 3 identified additional cancer-related genes in both DN and DE analyses. Our findings suggest that using TP as a covariate in a linear model is appropriate for DE analysis, but a more robust model is needed for DN analysis. However, because true DN or DE patterns are not known for the empirical datasets, simulated datasets can be used to study the statistical properties of these methods in future studies.

## Introduction

The tumor microenvironment (TME) is composed of tumor cells, stroma cells, immune cells, blood vessels, and other associated non-cancerous cells. It is recognized that TME is a key contributor to tumor growth, progression, and metastasis (Quail and Joyce, 2013; Turley et al., 2015). Advances in high-throughput sequencing technologies have enabled a comprehensive view of this heterogeneous collection of cells. The tumor purity (TP) is defined as the proportion of tumor cells in a solid tumor sample. TP is important to know because it contributes to a better prediction of prognosis and clinical management (Mao et al., 2018; Gong et al., 2020). It also plays a crucial role in classifying cancer subtypes (Zhang et al., 2017).

Conventionally, the TP is estimated through a visual inspection of tumor specimens between trained pathologists (Rajan et al., 2004), which can cause a poor interrater agreement and be time-consuming for large studies (Yuan et al., 2012; Haider et al., 2020). Researchers have been investigating the estimation of TP directly from data. Several studies have proposed methods of estimating the TP in DNA methylation data (updated version of InfiniumPurify; Zheng et al., 2017), DNA somatic copy number data (ABSOLUTE algorithm; Carter et al., 2012), high-throughput DNA-sequencing data (Tumor Heterogeneity Analysis algorithm; Oesper et al., 2013), and whole-exome sequencing data (AbsSN-Seq algorithm; Bao et al., 2014). Lastly, Yoshihara et al. (2013) developed the ESTIMATE (Estimation of STromal and Immune cells in MAlignant Tumor tissues using Expression data) algorithm for TP estimation in microarray data, which is based on a scoring system using the proportion of stromal and immune cells in tumor samples.

In this study, our interest lies in RNA-seq data. Research involving the estimation of TP from RNA-sequencing (RNA-seq) data was presented with the eXtreme Gradient Boosting (XGBoost) ensemble learning algorithm (Li et al., 2019) and with the gene co-expression network-based TSNet model (Petralia et al., 2018).

Beyond the estimation of TP, Aran et al. (2015) analyzed RNA-seq data across 21 cancer types from The Cancer Genome Atlas (TCGA; Cancer Genome Atlas Research Network et al., 2013) using the TP in their analyses. They examined the association between TP and clinical variables and differences in TP across different subtypes of cancer. Evidence from their studies indicates that the TP confounds the association between gene expression and overall survival (OS) in the differential expression (DE) analysis. They conducted the DE analysis across 13 types of cancer, then compared it to a similar analysis with the inclusion of purity estimates as an additional covariate. Genes that were initially DE between tumor and normal samples before adding TP as a covariate turn out not to be DE, and a set of new genes were introduced as DE after adding TP into the analysis (Aran et al., 2015). In another recent study, Rhee et al. (2018) performed the gene cluster analysis using a partial correlation to identify the relationship between the gene expression and mutation abundance while adjusting for TP.

However, there are a limited number of studies that assess the effect of TP on other statistical methods (Zhang et al., 2017; Petralia et al., 2018) that are widely used for analyzing gene expression data, such as differential network (DN) analysis. In this article, we have two main objectives. These will contrast results from three different analyses: analyzing the complete dataset without TP information (Analysis 1); analyzing the dataset after dichotomizing TP and removing the low-purity samples (Analysis 2); and analyzing the complete dataset with TP included as a continuous covariate (Analysis 3).

**FIGURE 1:**
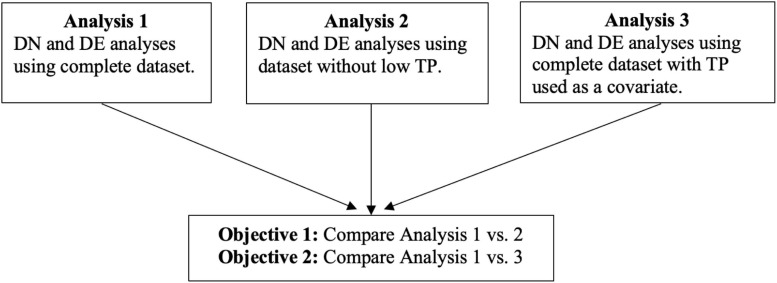
Flowchart of analysis plans and objectives of the study.

In the first objective, we compare results of Analysis 1 to Analysis 2. In the second objective, we compare results between Analysis 1 and Analysis 3. In both objectives, we analyzed breast invasive carcinoma (BRCA), head and neck squamous cell carcinoma (HNSC), and lung squamous cell carcinoma (LUSC) datasets from the TCGA (Cancer Genome Atlas Research Network et al., 2013). Figure 1 summarizes the analysis plans and objectives of the study. The approach described in this paper provides a general strategy for assessing the effect of TP on gene expression data analyses.

## Materials and Methods

### Clinical Data

An initial sample of 1,093 patients were obtained from the BRCA dataset. After exclusion of patients with incomplete data on age at diagnosis, OS, and TP, 1,029 patients remained eligible for the study. Similarly, 509 and 474 patients were used for analysis after excluding 11 and 27 patients from the HNSC and LUSC datasets, respectively. The primary endpoint was OS, calculated as the time from diagnosis to the time of death. Patients who were alive at the last follow-up were considered censored. The rate of censoring was 85.6, 58, and 57.8% for BRCA, HNSC, and LUSC.

### RNA-Seq Data

The normalized RNA-seq data consisting of 20,155 genes from TCGA for the breast cancer samples were obtained from LinkedOmics (Vasaikar et al., 2017), a publicly available portal that contains multi-omics data and clinical data across 32 cancer types from TCGA. For all analyses, genes without an Entrez gene ID were removed. A total of 16,485 genes were mapped to its Entrez gene ID. It was further reduced to 6,963 genes which were also found in 7,618 unique genes from Reactome pathways (Jassal et al., 2020). The Reactome database is an open-source and peer-reviewed database of biological pathways. To filter out lowly expressed genes, genes with zero Reads Per Kilobase of transcript per Million reads mapped (RPKM) expression in more than 80% of 1,029 samples were removed, leaving 6,747 genes. Upon applying the same data processing scheme, 6,698 out of original 20,165 genes from 509 samples and 6,712 out of original 20,104 genes from 474 patients were available for the analysis of HNSC and LUSC, respectively. We considered genes that are within 649 pathways (Supplementary Files for complete list) that have more than 20 or less than 100 genes for the analysis of DN.

### Statistical Methods

Our objective is to assess the effect of TP on DN analysis, which has not been studied previously, and on DE analysis by comparing Analysis 1 vs. Analysis 2 and Analysis 1 vs. Analysis 3. The methods to the analyses of DN and DE are described below. Study samples are dichotomized into high-survival (HS) and low-survival (LS) groups based on the median value of OS. All statistical analyses were performed in R version 4.0.2 (R Foundation for Statistical Computing, Vienna, Austria).

#### Differential Network Analysis

The DN analysis is a method for identifying changes among gene–gene associations. These changes are indicative of dysfunctional regulation that is affecting the ability of genes to interact with one another (either through their mRNA or protein products; de la Fuente, 2010). Genes do not work alone; in other words, they interact with each other in complicated ways. However, the DE analysis assumes that the gene expression is independent of each other, which lacks in identifying the dynamics of physical and genetic networks directly (Ideker and Krogan, 2012; Kim et al., 2018). DN analysis is different from DE analysis in that it compares a weighted network from study samples with different clinical characteristics to identify a set of genes involved in a specific cancer-related pathway or to find a hub gene that regulates its neighbor genes. The HS and LS groups are compared to identify gene pathways that have differentially connected (DC) co-expression networks. The *dnapath* package (Grimes et al., 2019) was used to perform the DN analysis using 649 different Reactome pathways, using partial correlations to infer the individual gene networks. The *p*-value of the differential connectivity score is computed from a permutation test (20 random permutations).

#### Differential Expression Analysis

The DE analysis was performed to identify the number of differentially expressed genes (DEGs) between HS and LS groups. The *edgeR* package (Robinson et al., 2010) was utilized to obtain the count matrix of gene counts. Subsequently, the gene-wise linear model is fitted to the data, followed by estimating contrasts of each gene using the *limma* package (Ritchie et al., 2015). Empirical Bayes smoothing was also applied to obtain the unadjusted gene-wise *p*-value. The Benjamini–Hochberg correction was then applied to control the false discovery rate for multiple-hypothesis testing.

#### Tumor Purity-Adjusted Analysis: Plans for Analysis 3

Tumor purity-adjusted analysis (Analysis 3) is compared to Analysis 1 to assess the confounding effect of TP on the association between gene expression and OS. We fit the simple linear regression model for each gene as a function of TP. The residual of each separate linear model is then utilized as TP-adjusted gene expression level for the TP-adjusted DN analysis. For the TP-adjusted DE analysis, TP is introduced as an additional covariate into the design matrix, as performed by Aran et al. (2015).

## Results

### Define High Tumor Purity and Survival Groups

**FIGURE 2:**
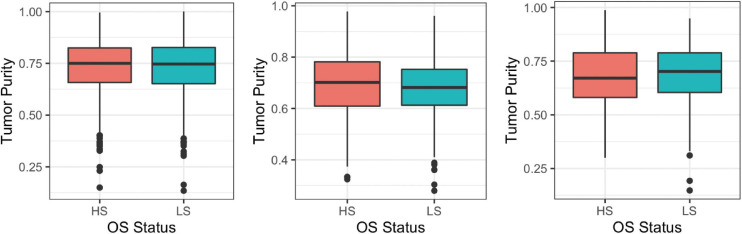
Boxplots of tumor purity (TP) between high and low overall survival (OS) groups, dichotomized based on the median OS using breast invasive carcinoma (BRCA; **left**), head and neck squamous cell carcinoma (HNSC; **center**), and lung squamous cell carcinoma (LUSC; **right**); HS, high overall survival; LS, low overall survival.

In order to compare results of Analysis 1 to Analysis 2 in later sections, we firstly need to define a cutoff value for “high” purity. The median TP from each three datasets is about 0.7 when rounding to the nearest 10th. Specifically, median (Q1, Q3) TPs for BRCA, HNSC, and LUSC are 0.747 (0.656, 0.825), 0.688 (0.613, 0.767), and 0.684 (0.590, 0.789), respectively. Therefore, it makes sense to treat TP greater than or equal to 0.7 as high purity. For DN and DE analyses, survival groups are dichotomized based on the median OS. Figure 2 displays boxplots of TP for two survival groups for the three cancer datasets.

### Analysis Without Tumor Purity Adjustment: Analysis 1 vs. 2

#### Differential Network Analysis on Three Cancer Types

The DN analysis was performed on the following study samples: full BRCA containing 1,029 samples (509 and 474 samples for full HNSC and full LUSC), and on a high-purity subset, which contained 659 samples (240 and 225 samples for HNSC and LUSC) after removing those with TP less than 0.70. The top five significant pathways from the DN analysis on BRCA are shown in Tables 1, 2 for Analysis 1 and Analysis 2, respectively. The top 20 significant pathways for Analyses 1 and 2 on BRCA are presented as Supplementary Tables 1, 2, respectively.

**TABLE 1:** Five most significant pathways from DN analysis using BRCA without subsetting.

**Pathway**	**DC score**	**No. of genes**	**No. of DC genes**	**Avg. expr. in low-risk**	**Avg. expr. in high-risk**
Inflammasomes	0.077	23	4	7.83	7.82
MET activates PTK2 signaling	0.075	30	3	10.2	10.2
Intrinsic pathway of fibrin clot formation	0.072	22	3	5.03	5.03
PD-1 signaling	0.072	23	3	7.12	7.09
Antigen activates B cell receptor (BCR) leading to generation of second messengers	0.072	32	4	8.98	8.93

*Columns include Reactome pathway names, differentially connectivity (DC) score, number of genes in the pathway, number of significant DC genes, and average expression level of genes in the pathway.*

**TABLE 2:** Five most significant pathways from DN analysis using BRCA subsetting on samples with tumor purity (TP) above 70%.

**Pathway**	**DC score**	**No. of genes**	**No. of DC genes**	**Avg. expr. in low-risk**	**Avg. expr. in high-risk**
G0 and early G1	0.086	27	3	8.97	8.89
Transcription of E2F targets under negative control by DREAM complex	0.085	19	5	9.31	9.24
Degradation of AXIN	0.081	55	6	10.3	10.3
SCF (Skp2)-mediated degradation of p27/p21	0.081	60	9	10.5	10.5
Cross-presentation of soluble exogenous antigens (endosomes)	0.08	50	3	9.79	9.8

*Columns include Reactome pathway names, DC score, number of genes in the pathway, number of significant DC genes, and average expression level of genes in the pathway.*

Among the top five results from Analysis 1 on BRCA (Table 1), four are non-tumor-related pathways: “Inflammasomes,” “PD-1 signaling,” and “antigen” are related to immune cells and “Fibrin clot formation” pathways are related to blood, except the “MET activates PTK2 signaling” pathway, which is related to the cell cycle. On the other hand, the top pathways from Analysis 2 (Table 2) are cancer-progression-related pathways, including cell cycle and transcription factor.

“Degradation of AXIN” is identified as one of the DC pathways in both analyses; in particular, it was the top 11th and 3rd in the full dataset and in the high-purity subset, respectively. AXIN is a protein that is related to a cytoskeletal regulation and a molecular controller of cerebral cortical development (Ye et al., 2015).

**FIGURE 3:**
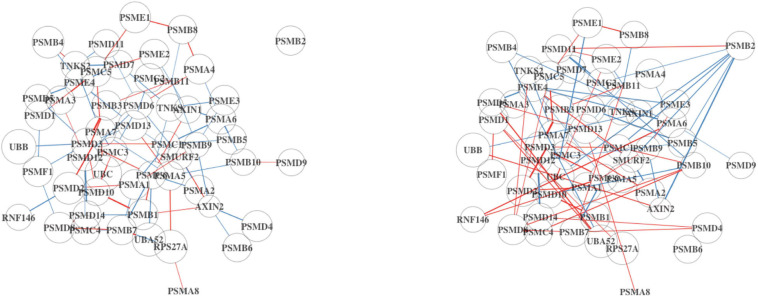
Differential network (DN) analysis results for the degradation of AXIN pathway using BRCA. On the left is the DN estimated from the full dataset, and on the right shows the estimated DN from the high TP subsample. The edge width and opacity are scaled based on (1) the *p*-value of the differential connectivity score and (2) the relative magnitude of the change in association. Blue edges indicate stronger association in the LS group, and red edges are stronger in the HS group. No connected edge between genes means that there is no statistical evidence of a gene–gene association. The edge color represents the relative mean gene expression for a specific grouping factor (HS and LS). The network will contain more disconnected components if the hub genes are no longer hubs, which potentially alter the network structure.

Incidentally, the mean expression of the “Degradation of AXIN” pathway is the same in both Analyses 1 and 2 (10.3 vs. 10.3), which we would not expect since the full dataset will contain more immune cells. However, the signal in the DN is stronger in Analysis 2 (Figure 3). Some of the edges (differential connections) are more prominent in Analysis 2 results. This suggests that the associations among genes in this pathway may be a result of dysregulation in the tumor cells rather than in the immune cells of the TME.

**FIGURE 4:**
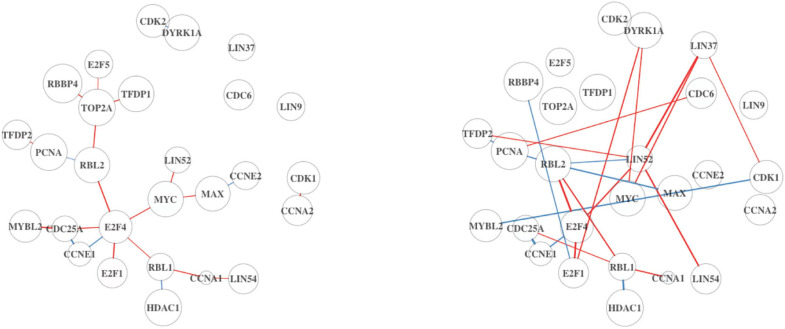
Differential network analysis results for the G0 and early G1 pathway using BRCA. On the left is the DN estimated from the full dataset, and on the right shows the estimated DN from the high TP subsample.

The “G0 and Early G1” pathway is significantly DC in Analysis 2, but not in Analysis 1. Upon inspection (Figure 4), we find that the two estimated DN show a greater difference compared to the previous comparison in Figure 3. This pathway is related to cell proliferation and may not be an active process within non-tumor cells in the TME. This would explain why the signal is weak in the full dataset. By subsetting on high-purity samples, the noise from the non-tumor cell in the TME is reduced.

Supplementary Table 3 summarizes the top 20 results of Analysis 1 on HNSC; of the top five pathways, three pathways are relevant to non-tumor cells in TME. Similar with BRCA, more cancer-progression-related pathways are found as top pathways in Analysis 2 on HNSC (Supplementary Table 4). However, based on the top five results of Analyses 1 and 2 on HNSC (Supplementary Tables 5, 6, respectively), there are four cancer-related pathways in Analysis 1 and three in Analysis 2. In all cancer datasets, the network plots (Supplementary Figures 1–3) for Analysis 2 shows greater amount of differential connectivity than Analysis 1.

#### Differential Expression Analysis on Three Cancer Types

A total of 6,747 genes are analyzed to identify DEGs between two survival groups in BRCA. One hundred seventy-seven genes are selected as DEGs between HS and LS groups in Analysis 1 (*n* = 1,029). Of these, 84 genes are upregulated and 93 genes are downregulated. Among the top five DEGs in Table 3, the Fc fragment of IgG receptor IIIa (FCGR3A) is linked to rheumatoid arthritis (Shimizu et al., 2019) and is associated with HIV infection (Poonia et al., 2010). Ribosomal protein (RPL22) plays a critical role in regulating lymphoma development (Rao et al., 2012). On the other hand, there is no DEG found (at the 0.05 significance level) between two survival groups in Analysis 2 (*n* = 659; Table 4).

**TABLE 3:** Five most significant differentially expressed genes (DEGs) from differential expression (DE) analysis using BRCA without subsetting.

**Gene**	**logFC**	**Avg. expr.**	**BH adj. *p*-value**
FCGR3A	0.33	10.5	0.006
RPL22	−0.16	12.7	0.006
SLCO2B1	0.30	9.6	0.006
RPS25	−0.19	12.7	0.006
SMPD1	0.18	9.5	0.006
			

**TABLE 4:** Results from DE analysis using BRCA subset on samples with TP above 70%.

**Gene**	**logFC**	**Avg. expr.**	**BH adj. *p*-value**
STAB1	0.29	9.4	0.062
SLCO2B1	0.31	9.1	0.102
RPS24	−0.24	14.1	0.102
RPL15	−0.16	14.0	0.111
HMGB1	−0.16	12.4	0.111

For HNSC, 755 out of 6,698 genes are DE between HS and LS groups in Analysis 1 (*n* = 509) whereas nine genes are DE in Analysis 2 (*n* = 240). The top five DEGs are summarized in Supplementary Tables 7, 8 for Analyses 1 and 2, respectively. For LUSC, there are 3 out of 6,712 genes identified as DEGs in Analysis 1 (*n* = 474), but no DEG is found in Analysis 2 (*n* = 225). Supplementary Tables 9, 10 list the top five DEGs from Analyses 1 and 2, respectively. Similar with BRCA, cancer-related genes are found among DEGs in HNSC and LUSC, which are shown in Supplementary Summary.

### Analysis With Tumor Purity Adjustment: Analysis 1 vs. 3

#### Tumor Purity-Adjusted Differential Network Analysis on Three Cancer Types

We further investigated the effect of TP by modeling it as a covariate. Previous studies have suggested that TP has a confounding effect on gene expression and conducted their analyses with TP adjustment (Aran et al., 2015; Rhee et al., 2018). Here, we perform TP-adjusted analyses of DN and DE (Analysis 3), and compare results with Analysis 1 in earlier sections.

**FIGURE 5:**
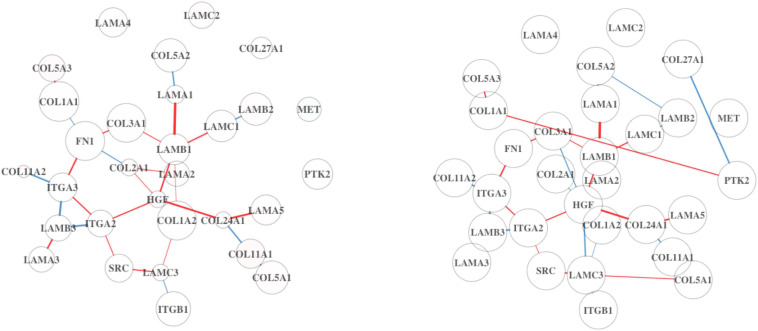
MET activates the PTK2 signaling pathway from DN analysis results using BRCA. On the left is the DN estimated from the full dataset not adjusted by TP, and on the right shows the estimated DN from the full dataset adjusted by TP.

**FIGURE 6:**
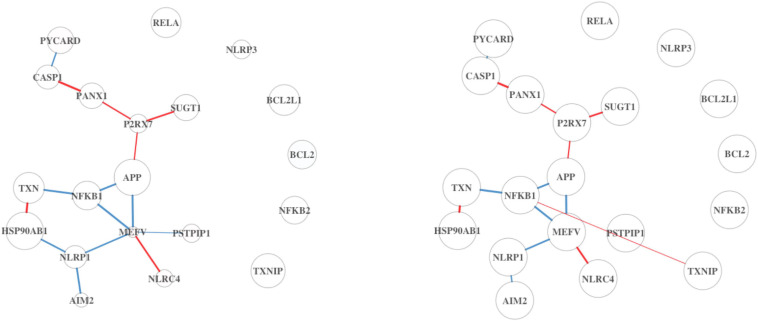
Inflammasome pathway from DN analysis results using BRCA. On the left is the DN estimated from the full dataset not adjusted by TP, and on the right shows the estimated DN from the full dataset adjusted by TP.

The top five pathways from Analysis 3 on BRCA (Table 5) resulted in a similar list of significant pathways compared to the ones from Analysis 1 (Table 1). The top 20 results are summarized in Supplementary Table 11; of these, “Listeria monocytogenes” is a pathogenic bacterium that has been studied for its use as cancer vaccines (Flickinger et al., 2018). ODC is an enzyme, whose overexpression is associated with the poorer OS in endometrial cancer (Kim et al., 2017). These two pathways are found as top 7th and 8th in Analysis 1 as well. Upon inspection of the first two significant pathways (Figures 5, 6), both analyses have similar network structures. However, some changes in differential connectivity are observed when adjusting for TP. For example, two edges that were not detected in Analysis 1 but appear in Analysis 3 include COL27A1-PTK2 and NFKB1-TXNIP in Figures 5, 6, respectively. PTK2 is linked to worse OS in ovarian and invasive breast cancer (Sulzmaier et al., 2014). Low expression in TXNIP is observed in different types of cancers including breast and stomach cancers (Nagaraj et al., 2018). These cancer-related DC genes may be useful for therapeutic development for cancer treatment, but should be carefully interpreted as these findings are estimates, not representing the true gene–gene association.

**TABLE 5:** Five most significant pathways from TP-adjusted DN analysis on BRCA.

**Pathway**	**DC score**	**No. of genes**	**No. of DC genes**	**Avg. expr. in low-risk**	**Avg. expr. in high-risk**
MET activates PTK2 signaling	0.076	30	2	−0.0311	0.0312
Inflammasomes	0.073	23	4	−0.00432	0.00434
PD-1 signaling	0.072	23	3	−0.00249	0.0025
Listeria monocytogenes entry into host cells	0.072	21	1	0.0235	−0.0237
Regulation of ornithine decarboxylase (ODC)	0.071	51	7	−0.00403	0.00405

*Columns include Reactome pathway names, DC score, number of genes in the pathway, number of significant DC genes, and average expression level of genes in the pathway.*

Supplementary Tables 12, 13 display the top 20 results of Analysis 3 on HNSC and LUSC, respectively. As shown in BRCA, Analysis 3 resulted in a similar list of pathways with Analysis 1. Upon the inspection of Supplementary Figures 5–7, the networks in Analyses 1 and 3 maintain a homogeneous structure with some minor differences, which we also observed in BRCA. Supplementary Summary further discusses findings about new edges detected on HNSC and LUSC.

#### Tumor Purity-Adjusted Differential Gene Expression Analysis on Three Cancer Types

Table 6 summarizes the top five DEGs found from Analysis 3 on BRCA. Two hundred forty-three out of 6,747 genes are DE between HS and LS groups. Among 243 DEGs, 125 genes are upregulated and 118 genes are downregulated. One hundred seventy-seven DEGs from Analysis 1 on BRCA are overlapped with these 243 DEGs found in Analysis 3. In addition, 66 DEGs are introduced in Analysis 3. Of 66 new DEGs, cytohesin 4 (CYTH4) is linked to bipolar disorder (Rezazadeh et al., 2015). Neutrophil cytosolic factor 4 (NCF4) is associated with the risk of colorectal cancer (Ryan et al., 2014). Triggering receptor expressed on myeloid cells 2 (TREM2) is related to Alzheimer’s disease development (Gratuze et al., 2018). Cyclin T2 (CCNT2) and acyl-CoA synthetase long-chain family member 5 (ACSL5) are involved with breast cancer (Stelzer et al., 2016). These findings about additional genes from Analysis 3 will facilitate research in understanding underlying mechanism of breast cancer.

**TABLE 6:** Five most significant DEGs from TP-adjusted DE analysis using BRCA.

**Gene**	**logFC**	**Avg. expr.**	**BH adj. *p*-value**
SLCO2B1	0.33	9.6	1.04e-06
FCGR3A	0.35	10.5	3.57e-05
C3AR1	0.27	8.3	3.57e-05
STAB1	0.28	9.7	3.57e-05
C1QC	0.29	10.6	3.58e-05

With HNSC, 615 out of 6,698 genes are found DE between HS and LS groups in Analysis 3. Six hundred two out of 615 DEGs overlap with DEGs from Analysis 1, and the remainder of 13 DEGs are detected in Analysis 3 only. The top five DEGs are summarized in Supplementary Table 14. For LUSC, 8 out of 6,712 genes are identified DE in Analysis 3; of these eight DEGs, five are found additionally and three overlap with DEGs from Analysis 1. Supplementary Table 15 displays the top five DEGs from Analysis 3. A cancer-related gene such as PLK3 is found DE. More details about HNSC and LUSC are discussed in Supplementary Summary. We have also included a complete list of genes and pathways that are identified from DE and DN analyses as Supplementary Files for each cancer types.

## Discussion

In this study, we assessed the effect of TP on DN and DE analyses by analyzing three RNA-seq datasets from TCGA. In both cases, qualitatively different results were obtained when filtering samples based on the TP or by including TP as a covariate.

For DN analysis, pathways related to immune and blood cells in TME were found in Analysis 1, while more cancer-related pathways were obtained in Analysis 2 except for LUSC. The same was not true for Analysis 3, which identified the same list of pathways as Analysis 1 in all three cancer datasets. This suggests that using TP as a covariate may not be sufficient for controlling its confounding effects on the association between gene expression and OS. Analysis 2 does not rely on any model assumptions, so it is more robust and may be able to identify the effect of TP. However, one limitation of Analysis 2 is that the decrease in sample size after removing low TP samples may influence the differences in results found when compared to Analysis 1.

For DE analysis, Analysis 1 revealed DEGs between HS and LS groups, while no or a few DEGs were identified in Analysis 2 in BRCA and HNSC. In LUSC, no or a few DEGs were found in either Analysis 1 or 2. When comparing Analysis 1 with Analysis 3, we observed similar results as in previous studies: adding TP as a covariate causes some DEGs to be removed while others are added. The linear model for the effect of TP on gene expression is reasonable for DE analysis, because we expect the aggregate gene expression of tumor-related genes to increase linearly as the ratio of tumor cells increases. Hence, Analysis 3 would have more power to detect the effect of TP on gene expression compared to the more robust approach of Analysis 2. By removing low TP samples, Analysis 2 is unable to utilize the full information provided by TP. However, results for DN analysis suggest that the linear model for TP is not the best choice in general. When comparing DEGs identified in our study to Aran et al., two genes are found in both studies using BRCA: TCF7 and MSR1. Sixteen DEGs are identified in both studies using HNSC: KCNA3, ABCD2, AQP1, FOXP1, C2orf49, PIK3CG, KDR, INPP5D, NFATC2, TNFAIP8L1, AVPR1A, MYO9B, F5, ARHGEF6, FBLN5, and ABCA6. However, there was no DEG overlapped with their studies using HNSC. This may be due to a different data processing scheme applied in each study.

We anticipate that our findings will lead to the improvement in understanding how to incorporate the TP when using two statistical methods: DN and DE analyses.

Future research could extend the current findings to examine how the TP-adjusted analysis affects the sensitivity and specificity compared to the unadjusted analysis. For example, we obtained more DEGs in BRCA and LUSC, but fewer DEGs in HNSC from the TP-adjusted DE analysis. In this paper, we did not include a simulation experiment on DN and DE analyses. It requires complex sampling methodology, which is beyond the scope of this paper. A possible simulation scenario is to set different model assumptions for gene expressions. For example, we consider a linear combination of gene expression level that is weighted by TP, and we also consider the null case when the gene expression level is independent from TP in which the linear combination assumption is not applied. DN and DE analyses can be performed using these simulated samples. Future studies are warranted focusing more on the effect of TP in a simulation-based study to validate our findings.

## Data Availability Statement

The datasets analyzed for this study can be available at www.linkedomics.org, further inquiries can be directed to the corresponding author.

## Author Contributions

SA, TG, and SD designed the study. SA and TG involved with the data processing and statistical analyses of the study. SA drafted the manuscript. TG and SD provided suggestions when writing the manuscript. All the authors have reviewed and edited the manuscript, contributed to the article and approved the submitted version.

## Conflict of Interest

The authors declare that the research was conducted in the absence of any commercial or financial relationships that could be construed as a potential conflict of interest.

## Publisher’s Note

All claims expressed in this article are solely those of the authors and do not necessarily represent those of their affiliated organizations, or those of the publisher, the editors and the reviewers. Any product that may be evaluated in this article, or claim that may be made by its manufacturer, is not guaranteed or endorsed by the publisher.
