# Supplementary material for: The Analysis of Gene Expression Data Incorporating Tumor Purity Information
Source: Front Genet. 2021 Aug 23;12:642759. doi: 10.3389/fgene.2021.642759 (PMC8419469; doi:10.3389/fgene.2021.642759)
Supplement: Supplementary file 4 [file Data_Sheet_4.docx]

Supplementary Material

# Supplementary Summary

# 1.1 Summary of DN Analysis on HNSC and LUSC for Analysis 1 vs. 2

Supplementary Table 3 summarizes the top 20 results of Analysis 1 on HNSC; of the top five pathways, three pathways are relevant to non-tumor cells in TME. “Molecules associated with elastic fibres,” “Regulation of Glucokinase,” and “interleukin signaling” are related to tissue cells, blood, and immune cells. On the other hand, “nuclear Pore Complex Disassembly” and “Transport of the SLBP independent Mature mRNA” pathways are related to cell cycle cancer-related pathways. SLBP is a cell cycle-regulated protein, discussed in the gastric cancer study [23-24]. Similar with BRCA, the top pathways such as “PRC2 methylates histones and DNA ” and “telomere maintenance” pathways from Analysis 2 in HNSC (Supplementary Table 4) are cancer-progression related pathways. In particular, previous findings have shown that mutations in PRC2 can contribute to cancer development and progression [25].

In addition, “synthesis of active ubiquitin: roles of E1 and E2 enzymes” pathway is another DC pathway in both analyses. Although the mean expression of the pathway between Analysis 1 and 2 was the same (10.6 vs. 10.6), Supplementary Figure 1 shows that the two networks have a different structure and Analysis 2 results have more edges with stronger signals. Ubiquitin is a regulatory protein, which is part of ubiquitin-proteasome system (UPS). Dysregulation of UPS can be seen in many types of cancer, and lead to tumor development and progression [26]. It is possible that these edges with stronger signals are masked by the non-tumor cell in the TME when not subsetting on high-purity samples.

In Supplementary Table 5, we summarize the top 20 results when Analysis 1 on LUSC. Among top four cancer-related pathways in the table, two pathways are involved with a displacement-loop (D-loop). D-loop is a non-coding region in mitochondrial DNA, and has been identified for the biomarker for early detection of head-and-neck cancer [27]. Syndecan, a transmembrane protein that is related to cancer initiation and development, is also found in Analysis 1 in LUSC. [28].

Based on top five pathways in Supplementary Table 6, three cancer-related pathways are found in Analysis 2: “Eukaryotic Translation Elongation,” “mRNA decay by 5' to 3' exoribonuclease,” and “ERCC6 (CSB) and EHMT2 (G9a) positively regulate rRNA expression.” These pathways are related to mRNA degradation, translation, and transcription factors. Of note, “ERCC6 (CSB) and EHMT2 (G9a) positively regulate rRNA expression” is found as one of the top DC cancer-related pathways in Analysis 1 as well. Supplementary Figure 2 shows that some edges in the two networks are differently colored according to the level of gene expression in each group, and new edges are introduced when analyzing the high-purity subset.

Another DC cancer-related pathway in both analyses is “Ubiquitin-dependent degradation of Cyclin D” pathway, related to cell cycle. Based on Supplementary Figure 3, two networks have a similar structure, but the network from Analysis 2 have increased number of edges. Similar with our findings from BRCA and HNSC, edges that are dormant with the greater presence of non-tumor cells in the TME become active when we focus on high-purity samples only. This suggests that presence of low TP may be an important confounder in gene expression analysis.

# 1.2 Summary of DE Analysis on HNSC and LUSC for Analysis 1 vs. 2

755 out of 6,698 total genes are DE between HS and LS groups in Analysis 1 using HNSC (n = 509). Among 755 DEGs, 454 genes are up-regulated, whereas 301 genes are down-regulated. In contrast, there are 9 DEGs only in Analysis 2 (n = 240). Of them, dpy-30 histone methyltransferase complex regulatory subunit (DPY30) is strongly associated with the development and migration of epithelial ovarian cancer [32]. Overexpression of aminoacyl tRNA synthetase complex interacting multifunctional protein 2 (AIMP2) is related to the proliferation of nasopharyngeal cancer cells [33]. All of these nine DEGs identified in Analysis 2 are also detected in Analysis 1. A small number of detection in DEGs may be due to a smaller sample size utilized in the analysis. Supplementary Table 7 and 8 summarize the top five DEGs in each analyses.

For DE analysis of LUSC, only 3 out of 6,712 genes are identified as DEGs between two survival groups in Analysis 1 (n = 474). Supplementary Table 9 and 10 summarized DE analysis results in each analyses. The three DEGs were all up-regulated. SIGIRR is critical in controlling intestinal inflammation and colitis-associated cancer [34] None of the genes are DE in Analysis 2 (n = 225). This is possibly due to a small sample size in each survival groups after subsetting. Also, this may suggest that two survival groups in LUSC have a similar clinical background.

# 1.3 Summary of DN Analysis on HNSC and LUSC for Analysis 1 vs. 3

Supplementary Table 12 displays the top 20 pathways from Analysis 3 on HNSC. Of them, “Regulation of Glucokinase by Glucokinase Regulatory Protein” pathway, a significant blood-related pathway obtained in Analysis 1 (Supplementary Table 3) is no longer DC after accounting for TP. “mTORC1-mediated signaling” pathway is involved in cell growth and mRNA translation [39]. Based on the two figures of tissue-related pathway (Supplementary Figure 4) and cell cycle-related pathway (Supplementary Figure 5), the overall structure of the networks in Analysis 1 and 3 look homogeneous but with some minor difference, due to TP-adjustment. While some edges that were present in Analysis 1 are not found, we detect new edges in Analysis 3, such as between ITGA8-EFEMP1 and NUP43-SEH1L in Fig. 10 and 11, respectively. ITGA8 is known to be linked with multiple myeloma progression [40]. According to GeneCards [41], NUP43 is associated with a rare genetic disorder leading to bone marrow failure, and SEH1L is related to nemaline myopathy. The analysis with TP adjustment enables to identify additional genes that can be help to further understand the comorbidities of HNSC.

We summarized our top 20 pathways from Analysis 3 using LUSC (Supplementary Table 13). As demonstrated with BRCA and HNSC, Analysis 3 produces a list of pathways, similar to Analysis 1 (Supplementary Table 5). An immune-related “Generation of second messenger molecules” pathway is introduced as significant pathway when TP-adjusted gene expression is used for Analysis 3. Among top four cancer-related pathways, two are selected to compare the differential network structure between Analyses 1 and 3 (Supplementary Figures 6 and 7). In general, both networks in each pathway maintain significant edges while a few disappear (e.g. VTN-SDC1 and PAK2-PSMC6 in Supplementary Figures 6 and 7, respectively). However, it is worth mentioning that some genes are DC when TP is accounted in the analysis. The following are the examples of new edges detected in “Syndecan interactions” pathway in Analysis 3: SDC4-FGF2, ITGAV-CASK, and COL3A1-SDC3 in Supplementary Figure 6. Of these genes, FGF2 may be a factor for gastric cancer development [42]. SDC4 and SDC 3 are related to thyroid carcinoma and Bardet-Biedl Syndrome, a rare genetic disorder, respectively [41]. “Ubiquitin-dependent degradation of Cyclin D” pathway is related to ubiquitin protein, which is also part of UPS. Hence, it is expected to observe that proteasome-related genes (PSMB and PSMD) have different magnitude of differential connectivity between each other in Analysis 3: PSMB1-PSMD7, PSMB11-PSMD7, PSMB10-PSMB1, and PSMB9-PSMD1 in Supplementary Figure 7. PSMB1 is linked to type I diabetes, and PSMD7 is related to Huntington’s disease and tracheitis [41].

# 1.4 Summary of DE Analysis on HNSC and LUSC for Analysis 1 vs. 3

In Analysis 3 on HNSC, 615 out of 6698 genes are DE between two survival groups. Supplementary Table 14 lists top five significant DEGs from this adjusted DE analysis. Among these 615 DEGs, 376 DEGs are up-regulated while 239 DEGs are down-regulated. Previously, we showed that 755 DEGs were found in Analysis 1. Of them, 602 DEGs genes are also remained DE between two survival groups in Analysis 3. 13 additional genes are discovered. According to the GeneCards database, makorin ring finger protein 1 (MKRN1) is associated with pilocytic astrocytoma, and mutation in the xanthine dehydrogenase (XDH) is found to cause xanthinuria, which affects the kidneys [41]. Unlike with BRCA findings above, TP adjustment yielded fewer number of DEGs than unadjusted analysis on HNSC.

For the Analysis 3 on LUSC, only 8 out of 6712 genes are found DE between HS and LS groups. Top five DEGs are summarized in Supplementary Table 15. Among 8 DEGs, 7 genes are up-regulated, and 1 gene is down-regulated. All of 3 DEGs obtained from Analysis 1 can also be seen in Analysis 3. Furthermore, 5 additional genes are observed DE in Analysis 3, which includes LCLAT1, CYBA, CEBPB, PLK3, and ADAMTSL4. Of these DEGs, recent work addressed that polo-like kinase 3 (PLK3) contributes to the regulation of cell cycle and found in many cancers [46] including breast cancer [41]. Mutations in ADAMTS-like protein 4 (ADAMTSL4) can cause autosomal recessive ectopia lentis, a displacement of crystalline lens [47]. As previously noted in Analysis 1, a small number of DEGs are obtained, possibly due to a small sample size in LUSC.

# Supplementary Figures


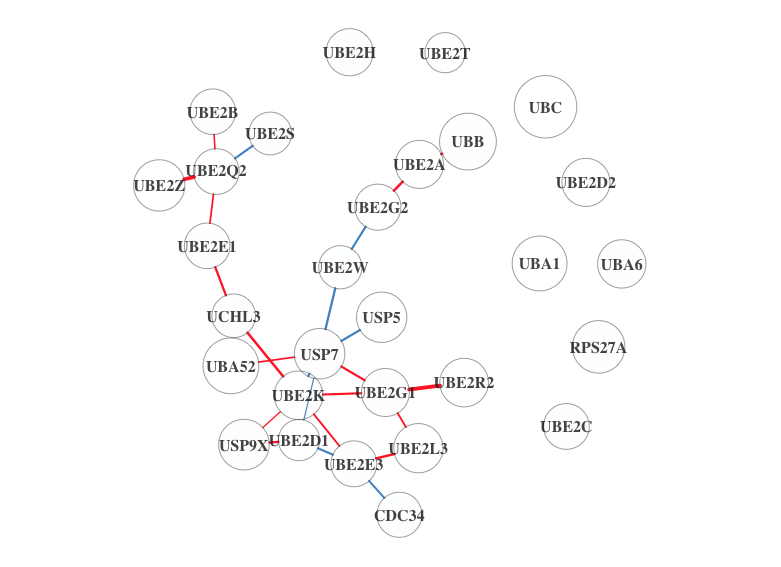

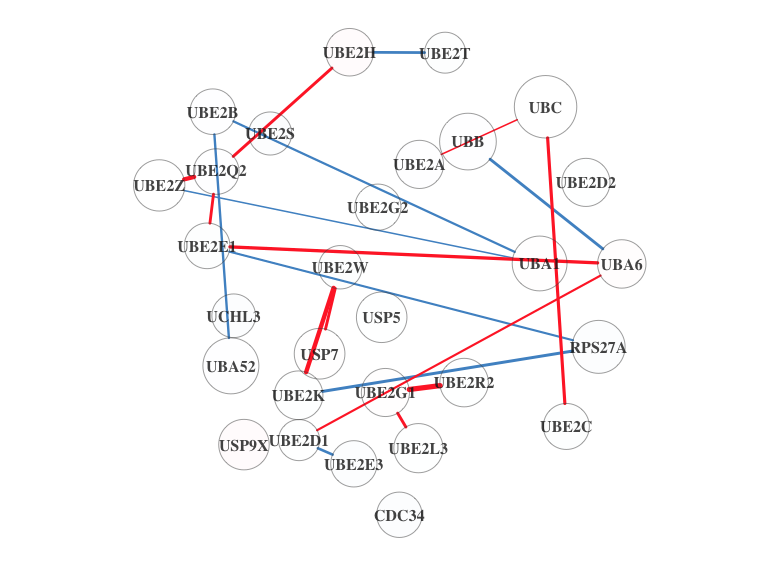


**Supplementary Figure 1.** Differential network analysis results for the synthesis of active ubiquitin: roles of E1 and E2 enzymes pathway using HNSC. On the left is the differential network estimated from the full dataset, and on the right shows the estimated differential network from the high tumor purity subsample.


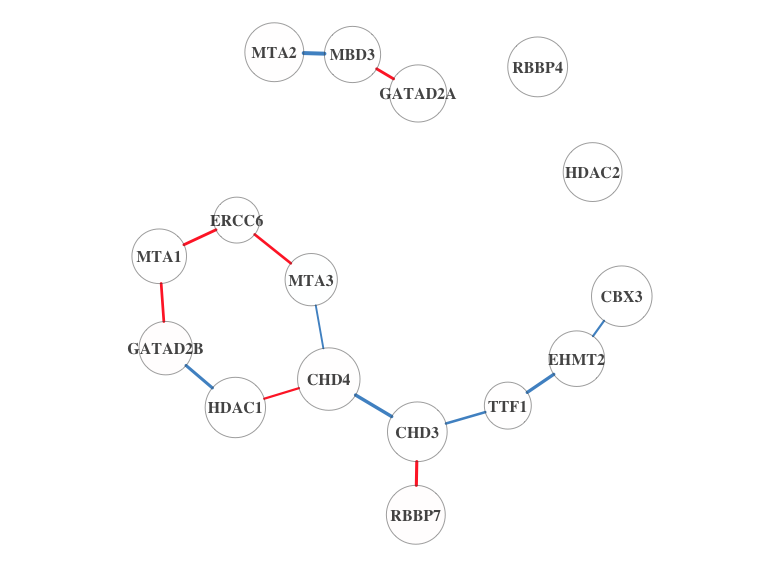

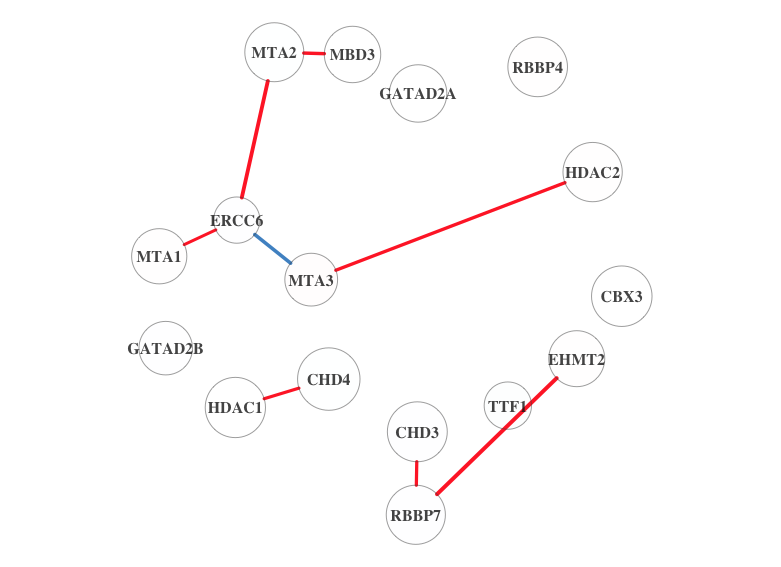


**Supplementary Figure 2.** Differential network analysis results for the ERCC6 (CSB) and EHMT2 (G9a) positively regulate rRNA expression pathway using LUSC. On the left is the differential network estimated from the full dataset, and on the right shows the estimated differential network from the high tumor purity subsample.


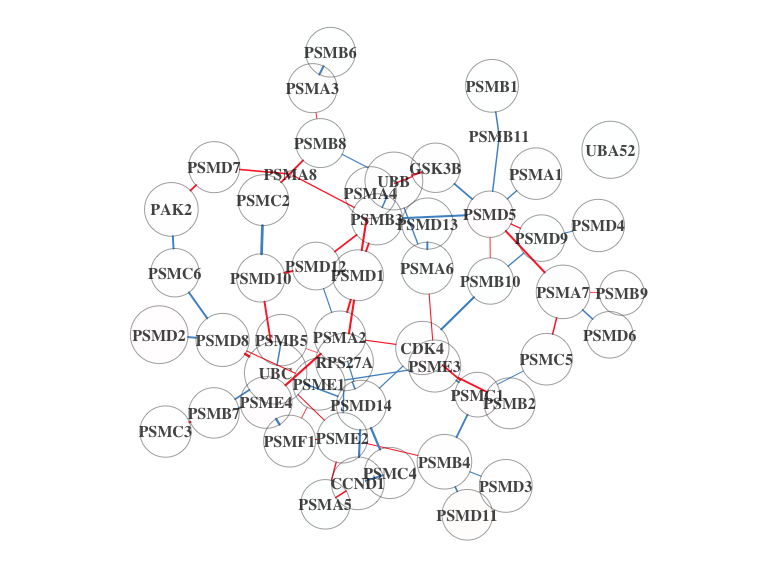

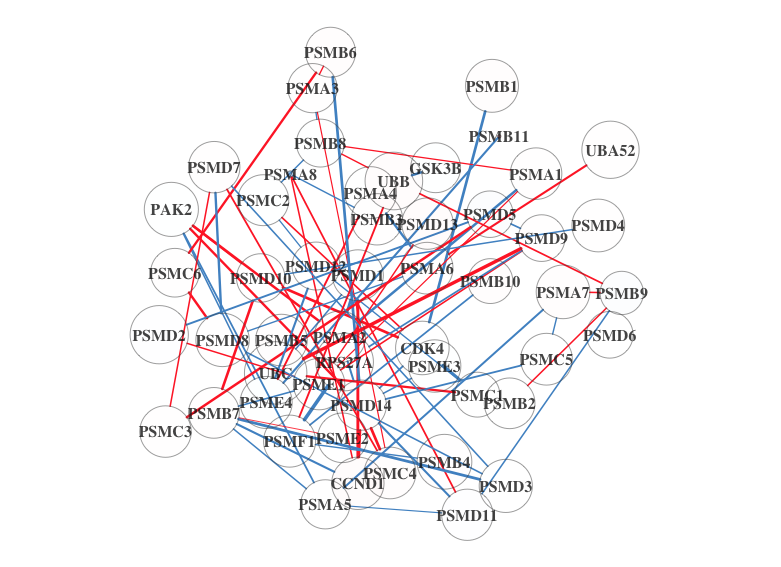


**Supplementary Figure 3.** Differential network analysis results for the Ubiquitin-dependent degradation of Cyclin D pathway using LUSC. On the left is the differential network estimated from the full dataset, and on the right shows the estimated differential network from the high tumor purity subsample.


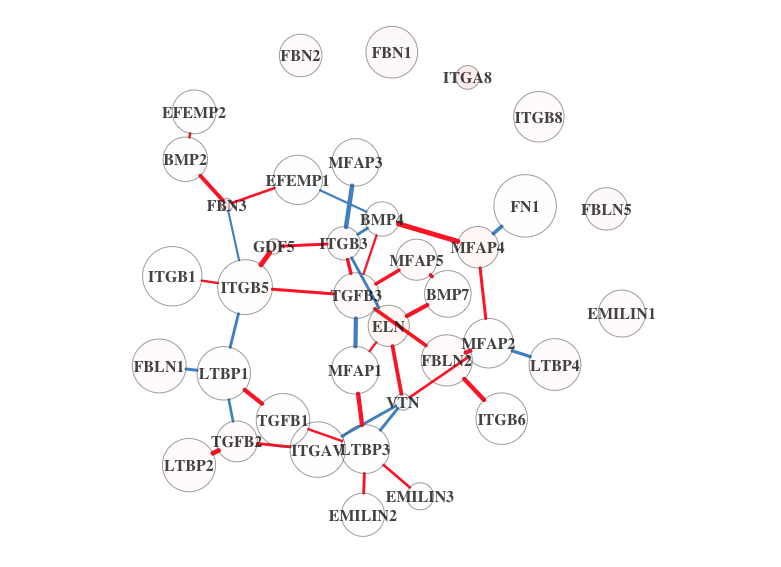

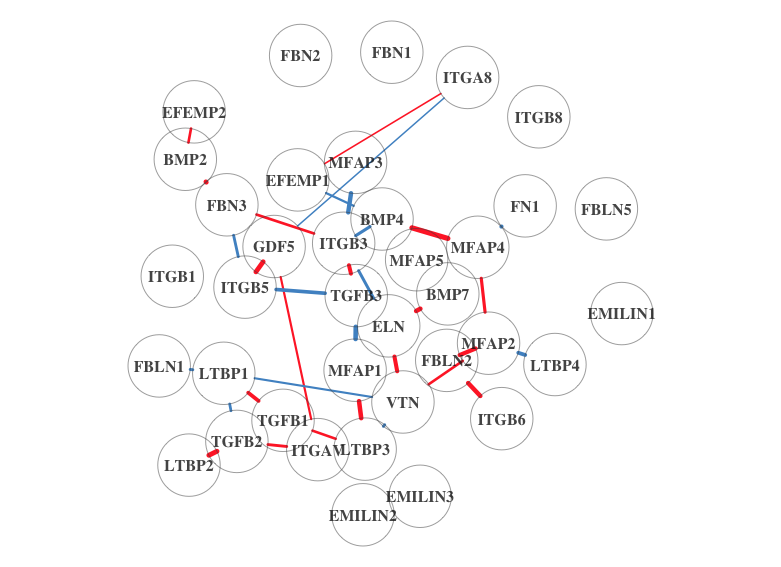


**Supplementary Figure 4.** Molecules associated with elastic fibres pathway from differential network analysis results using HNSC. On the left is the differential network estimated from the full dataset not adjusted by TP, and on the right shows the estimated differential network from the full dataset adjusted by TP.


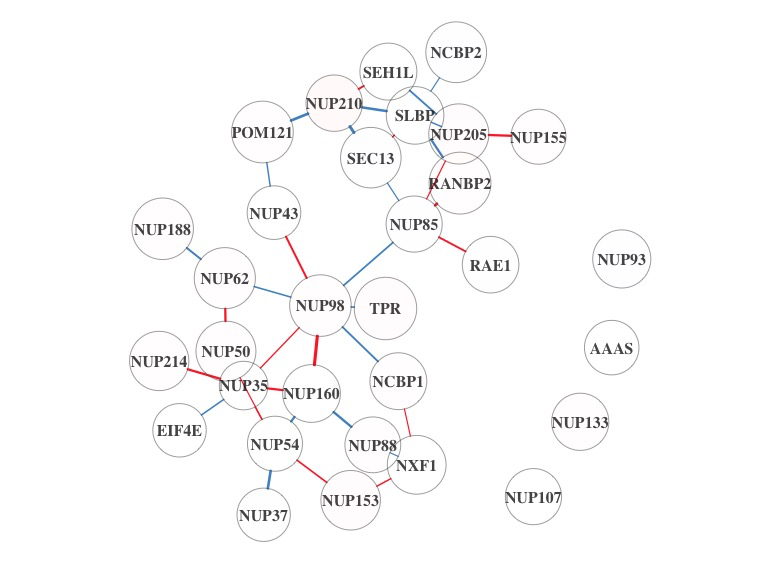

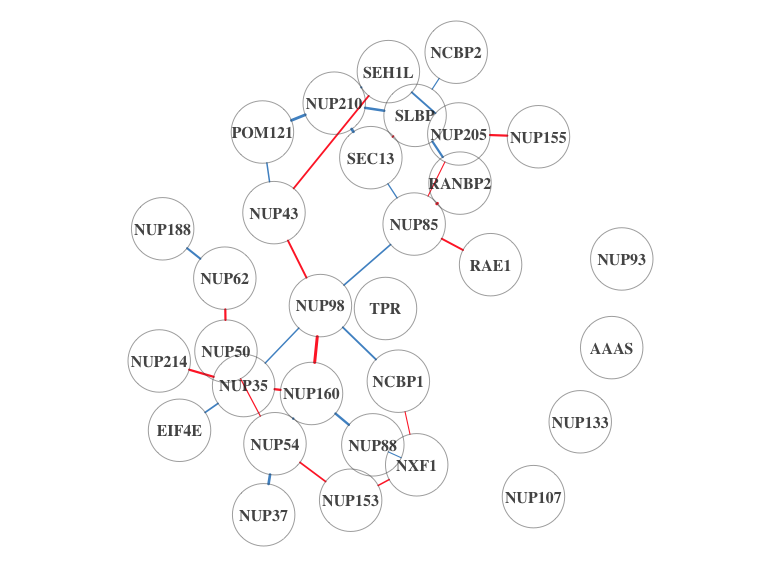


**Supplementary Figure 5.** Transport of the SLBP independent Mature mRNA pathway from differential network analysis results using HNSC. On the left is the differential network estimated from the full dataset not adjusted by TP, and on the right shows the estimated differential network from the full dataset adjusted by TP.


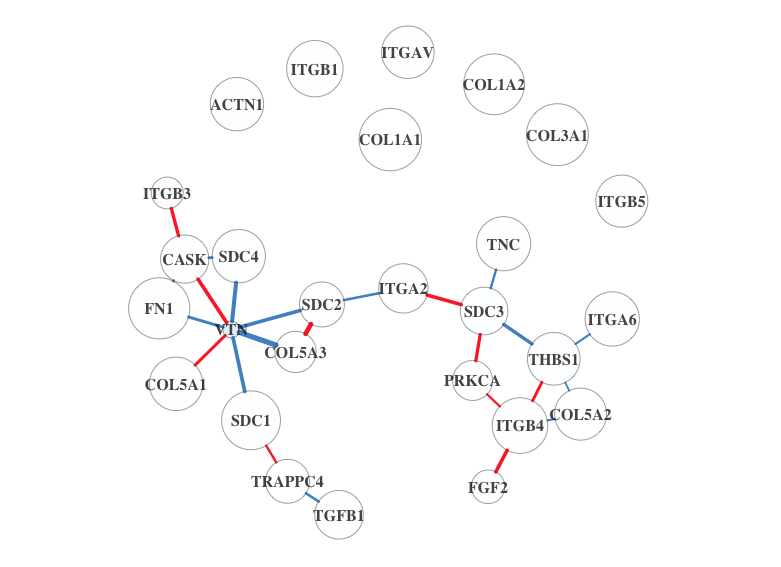

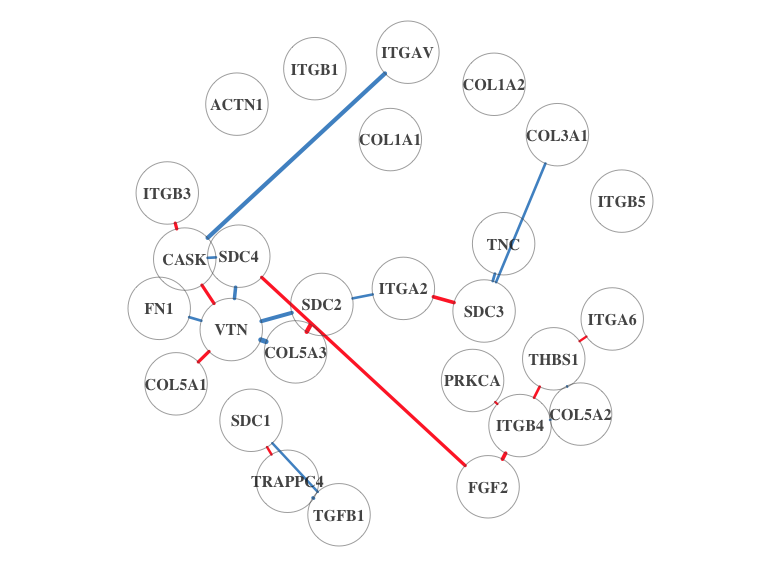


**Supplementary Figure 6.** Syndecan interactions pathway from differential network analysis results using LUSC. On the left is the differential network estimated from the full dataset not adjusted by TP, and on the right shows the estimated differential network from the full dataset adjusted by TP.


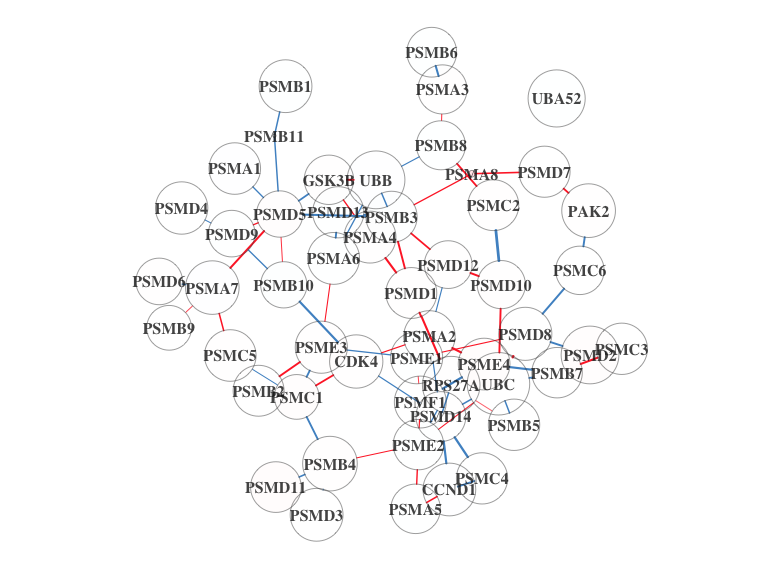

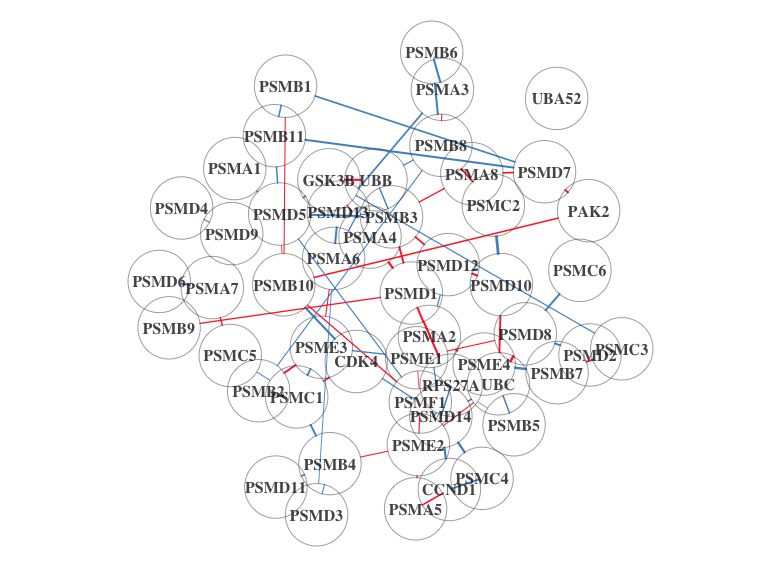


**Supplementary Figure 7.** Ubiquitin-dependent degradation of Cyclin D pathway from differential network analysis results using LUSC. On the left is the differential network estimated from the full dataset not adjusted by TP, and on the right shows the estimated differential network from the full dataset adjusted by TP.

# Supplementary Tables

| Pathway | DC score | # genes | # DC genes | Avg. expr.  in low-risk | Avg. expr.  in high-risk |
| --- | --- | --- | --- | --- | --- |
| Inflammasomes | 0.0765 | 23 | 4 | 7.83 | 7.82 |
| MET activates PTK2 signaling | 0.0748 | 30 | 3 | 10.2 | 10.2 |
| Intrinsic Pathway of Fibrin Clot Formation | 0.072 | 22 | 3 | 5.03 | 5.03 |
| PD-1 signaling | 0.0716 | 23 | 3 | 7.12 | 7.09 |
| Antigen activates B Cell Receptor (BCR) leading to generation of second messengers | 0.0716 | 32 | 4 | 8.98 | 8.93 |
| Eukaryotic Translation Elongation (See also: Peptide chain elongation) | 0.0713 | 93 | 12 | 12.1 | 12 |
| Listeria monocytogenes entry into host cells | 0.0712 | 21 | 1 | 9.89 | 9.84 |
| Regulation of ornithine decarboxylase (ODC) | 0.0711 | 51 | 8 | 10.2 | 10.2 |
| Ubiquitin-dependent degradation of Cyclin D (See also: Regulation of activated PAK-2p34 by proteasome mediated degradation) | 0.0708 | 53 | 8 | 10.5 | 10.5 |
| Complex I biogenesis | 0.0707 | 55 | 7 | 8.11 | 8.12 |
| Degradation of AXIN | 0.0707 | 55 | 8 | 10.3 | 10.3 |
| NOTCH4 Intracellular Domain Regulates Transcription | 0.0707 | 20 | 1 | 9.04 | 8.99 |
| Signal transduction by L1 | 0.0707 | 21 | 3 | 9.5 | 9.5 |
| Regulation of Apoptosis (See also: Regulation of activated PAK-2p34 by proteasome mediated degradation) | 0.0705 | 53 | 5 | 10.4 | 10.4 |
| Collagen chain trimerization | 0.0704 | 44 | 3 | 7.96 | 7.99 |
| Negative regulation of NOTCH4 signaling | 0.0703 | 54 | 7 | 10.8 | 10.8 |
| SCF(Skp2)-mediated degradation of p27/p21 | 0.0702 | 60 | 11 | 10.5 | 10.5 |
| activated TAK1 mediates p38 MAPK activation | 0.07 | 24 | 3 | 9.71 | 9.69 |
| Vif-mediated degradation of APOBEC3G | 0.0699 | 55 | 5 | 9.8 | 9.8 |
| RNA Polymerase III Transcription Initiation From Type 1 Promoter | 0.0699 | 28 | 4 | 9.48 | 9.48 |

**Supplementary Table 1.** Top 20 most significant pathways from differential network analysis using BRCA without subsetting. Columns include Reactome pathway names, differentially connectivity (DC) score, number of genes in the pathway, number of significant DC genes, and average expression level of genes in the pathway.

| Pathway | DC score | # genes | # DC genes | Avg. expr.  in low-risk | Avg. expr.  in high-risk |
| --- | --- | --- | --- | --- | --- |
| G0 and Early G1 | 0.0856 | 27 | 3 | 8.97 | 8.89 |
| Transcription of E2F targets under negative control by DREAM complex | 0.0854 | 19 | 5 | 9.31 | 9.24 |
| Degradation of AXIN | 0.0812 | 55 | 6 | 10.3 | 10.3 |
| SCF(Skp2)-mediated degradation of p27/p21 | 0.0807 | 60 | 9 | 10.5 | 10.5 |
| Cross-presentation of soluble exogenous antigens (endosomes) | 0.0801 | 50 | 3 | 9.79 | 9.8 |
| Regulation of RUNX3 expression and activity | 0.0801 | 55 | 5 | 10.6 | 10.6 |
| Regulation of Apoptosis (See also: Regulation of activated PAK-2p34 by proteasome mediated degradation) | 0.0799 | 53 | 5 | 10.4 | 10.4 |
| Degradation of DVL | 0.0796 | 57 | 4 | 10.2 | 10.2 |
| Hh mutants that don't undergo autocatalytic processing are degraded by ERAD (See also: Hh mutants abrogate ligand secretion) | 0.0794 | 59 | 5 | 10 | 10 |
| Dectin-1 mediated noncanonical NF-kB signaling (See also: NIK-->noncanonical NF-kB signaling) | 0.0794 | 60 | 3 | 10.3 | 10.3 |
| SMAD2/SMAD3:SMAD4 heterotrimer regulates transcription | 0.0794 | 32 | 5 | 9.85 | 9.79 |
| ZBP1(DAI) mediated induction of type I IFNs | 0.0789 | 21 | 0 | 8.75 | 8.76 |
| Insertion of tail-anchored proteins into the endoplasmic reticulum membrane | 0.0789 | 22 | 2 | 8.62 | 8.64 |
| FCERI mediated MAPK activation | 0.0784 | 32 | 6 | 9.34 | 9.32 |
| RNA Polymerase II Transcription Termination | 0.0783 | 67 | 6 | 7.79 | 7.77 |
| FCERI mediated Ca+2 mobilization | 0.0783 | 33 | 8 | 8.8 | 8.78 |
| Notch-HLH transcription pathway | 0.0778 | 28 | 3 | 9.75 | 9.75 |
| Post-chaperonin tubulin folding pathway | 0.0776 | 22 | 3 | 7.45 | 7.43 |
| AUF1 (hnRNP D0) binds and destabilizes mRNA | 0.0774 | 56 | 5 | 10.8 | 10.8 |
| Hedgehog ligand biogenesis | 0.0771 | 65 | 4 | 9.76 | 9.75 |

**Supplementary Table 2.** Top 20 most significant pathways from differential network analysis using BRCA subsetting on samples with tumor purity above 70%. Columns include Reactome pathway names, differentially connectivity (DC) score, number of genes in the pathway, number of significant DC genes, and average expression level of genes in the pathway.

| Pathway | DC score | # genes | # DC genes | Avg. expr.  in low-risk | Avg. expr.  in high-risk |
| --- | --- | --- | --- | --- | --- |
| Molecules associated with elastic fibres | 0.0928 | 38 | 5 | 8.67 | 8.82 |
| Regulation of Glucokinase by Glucokinase Regulatory Protein (See also: Defective TPR may confer susceptibility towards thyroid papillary carcinoma (TPC)) | 0.0913 | 31 | 4 | 8.73 | 8.76 |
| Other interleukin signaling | 0.0897 | 24 | 2 | 8.08 | 8.12 |
| Nuclear Pore Complex (NPC) Disassembly | 0.0893 | 35 | 7 | 9.35 | 9.37 |
| Transport of the SLBP independent Mature mRNA (See also: Transport of the SLBP Dependant Mature mRNA) | 0.0891 | 35 | 6 | 9.08 | 9.11 |
| Activation of SMO | 0.0881 | 18 | 4 | 6.59 | 6.66 |
| HDR through Single Strand Annealing (SSA) | 0.088 | 37 | 3 | 8.04 | 8.08 |
| Digestion and absorption | 0.0878 | 27 | 2 | 2.14 | 2.15 |
| SHC1 events in ERBB2 signaling | 0.0876 | 22 | 6 | 8.02 | 8.03 |
| Export of Viral Ribonucleoproteins from Nucleus (See also: NEP/NS2 Interacts with the Cellular Export Machinery) | 0.0874 | 39 | 5 | 8.13 | 8.15 |
| VEGFR2 mediated cell proliferation | 0.0867 | 21 | 1 | 10.3 | 10.3 |
| Transport of Mature mRNA Derived from an Intronless Transcript | 0.0861 | 42 | 5 | 9.3 | 9.31 |
| FRS-mediated FGFR1 signaling | 0.0861 | 23 | 1 | 4.91 | 4.97 |
| Apoptotic factor-mediated response | 0.086 | 18 | 2 | 9.08 | 9.08 |
| RA biosynthesis pathway | 0.0851 | 22 | 4 | 6.28 | 6.36 |
| Host Interactions with Influenza Factors (See also: NS1 Mediated Effects on Host Pathways) | 0.0848 | 45 | 5 | 8.88 | 8.89 |
| Chondroitin sulfate/dermatan sulfate metabolism | 0.0844 | 50 | 4 | 8.36 | 8.41 |
| Nuclear Envelope Breakdown | 0.0823 | 52 | 7 | 9.1 | 9.12 |
| PERK regulates gene expression | 0.0818 | 32 | 2 | 9.39 | 9.34 |
| Synthesis of active ubiquitin: roles of E1 and E2 enzymes | 0.0815 | 30 | 4 | 10.6 | 10.5 |

**Supplementary Table 3.** Top 20 most significant pathways from differential network analysis using HNSC without subsetting. Columns include Reactome pathway names, differentially connectivity (DC) score, number of genes in the pathway, number of significant DC genes, and average expression level of genes in the pathway.

| Pathway | DC score | # genes | # DC genes | Avg. expr.  in low-risk | Avg. expr.  in high-risk |
| --- | --- | --- | --- | --- | --- |
| PRC2 methylates histones and DNA | 0.13 | 73 | 3 | 1.72 | 1.73 |
| Initial triggering of complement | 0.114 | 23 | 4 | 5.56 | 5.7 |
| Telomere Maintenance | 0.11 | 82 | 4 | 4.17 | 4.15 |
| G0 and Early G1 | 0.11 | 27 | 2 | 9.42 | 9.42 |
| Abortive elongation of HIV-1 transcript in the absence of Tat | 0.108 | 23 | 4 | 8.63 | 8.55 |
| Mitochondrial calcium ion transport | 0.105 | 23 | 2 | 6.79 | 6.74 |
| Regulation of FZD by ubiquitination | 0.102 | 21 | 2 | 8.12 | 8.23 |
| Interleukin-20 family signaling | 0.1 | 25 | 2 | 6.56 | 6.6 |
| RA biosynthesis pathway | 0.1 | 22 | 4 | 6.31 | 6.42 |
| Digestion and absorption | 0.0994 | 27 | 1 | 2.11 | 2.11 |
| DAP12 interactions | 0.0986 | 43 | 4 | 6.71 | 6.82 |
| Meiotic synapsis | 0.0984 | 79 | 6 | 3.25 | 3.28 |
| FGFR2 alternative splicing | 0.0972 | 26 | 1 | 10.5 | 10.4 |
| Myogenesis | 0.0957 | 30 | 3 | 7 | 7.04 |
| Synthesis of active ubiquitin: roles of E1 and E2 enzymes | 0.0932 | 30 | 3 | 10.6 | 10.5 |
| PKMTs methylate histone lysines | 0.091 | 71 | 2 | 4.44 | 4.47 |
| DNA Damage/Telomere Stress Induced Senescence | 0.0891 | 80 | 5 | 3.28 | 3.29 |
| Sulfur amino acid metabolism | 0.0888 | 28 | 5 | 7.43 | 7.43 |
| The canonical retinoid cycle in rods (twilight vision) | 0.0872 | 23 | 2 | 5.23 | 5.15 |
| Formation of Incision Complex in GG-NER | 0.0864 | 43 | 3 | 10.2 | 10.1 |

**Supplementary Table 4.** Top 20 most significant pathways from differential network analysis using HNSC subsetting on samples with tumor purity above 70%. Columns include Reactome pathway names, differentially connectivity (DC) score, number of genes in the pathway, number of significant DC genes, and average expression level of genes in the pathway.

| Pathway | DC score | # genes | # DC genes | Avg. expr.  in low-risk | Avg. expr.  in high-risk |
| --- | --- | --- | --- | --- | --- |
| Resolution of D-loop Structures through Synthesis-Dependent Strand Annealing (SDSA) | 0.0955 | 26 | 5 | 7.25 | 7.32 |
| Syndecan interactions | 0.095 | 27 | 4 | 11.4 | 11.3 |
| Resolution of D-Loop Structures (See also: Resolution of D-loop Structures through Holliday Junction Intermediates) | 0.0919 | 34 | 3 | 6.41 | 6.46 |
| ERCC6 (CSB) and EHMT2 (G9a) positively regulate rRNA expression | 0.0916 | 77 | 2 | 2.25 | 2.25 |
| Pre-NOTCH Processing in Golgi | 0.091 | 18 | 2 | 9.97 | 9.93 |
| Platelet calcium homeostasis | 0.0909 | 30 | 5 | 7.06 | 7.01 |
| Role of phospholipids in phagocytosis | 0.0904 | 25 | 6 | 8.51 | 8.48 |
| Meiotic recombination | 0.09 | 86 | 4 | 2.17 | 2.18 |
| FGFR2 alternative splicing | 0.0854 | 26 | 3 | 10.5 | 10.5 |
| Interferon gamma signaling | 0.0846 | 92 | 9 | 8.78 | 8.72 |
| CD28 co-stimulation | 0.0844 | 33 | 4 | 9.35 | 9.34 |
| Ubiquitin-dependent degradation of Cyclin D (See also: Regulation of activated PAK-2p34 by proteasome mediated degradation) | 0.084 | 53 | 2 | 10.8 | 10.8 |
| Synthesis of PC | 0.0839 | 28 | 4 | 8.54 | 8.53 |
| PI-3K cascade:FGFR2 | 0.0838 | 23 | 4 | 4.49 | 4.49 |
| Regulation of Apoptosis (See also: Regulation of activated PAK-2p34 by proteasome mediated degradation) | 0.0831 | 53 | 2 | 10.6 | 10.6 |
| Defective CFTR causes cystic fibrosis | 0.0819 | 61 | 8 | 10.6 | 10.6 |
| Common Pathway of Fibrin Clot Formation | 0.0818 | 22 | 3 | 4.99 | 4.76 |
| Degradation of DVL | 0.0817 | 57 | 5 | 10.5 | 10.5 |
| Processing of Capped Intronless Pre-mRNA | 0.0817 | 28 | 0 | 10.1 | 10.2 |
| Metabolism of polyamines | 0.0812 | 59 | 5 | 10.2 | 10.2 |

**Supplementary Table 5.** Top 20 most significant pathways from differential network analysis using LUSC without subsetting. Columns include Reactome pathway names, differentially connectivity (DC) score, number of genes in the pathway, number of significant DC genes, and average expression level of genes in the pathway.

| Pathway | DC score | # genes | # DC genes | Avg. expr.  in low-risk | Avg. expr.  in high-risk |
| --- | --- | --- | --- | --- | --- |
| Eukaryotic Translation Elongation (See also: Peptide chain elongation) | 0.114 | 93 | 4 | 12.3 | 12.3 |
| mRNA decay by 5' to 3' exoribonuclease | 0.11 | 15 | 1 | 9.74 | 9.77 |
| Sema4D induced cell migration and growth-cone collapse | 0.107 | 21 | 5 | 11.2 | 11.2 |
| ERCC6 (CSB) and EHMT2 (G9a) positively regulate rRNA expression | 0.102 | 77 | 3 | 2.27 | 2.26 |
| Antigen activates B Cell Receptor (BCR) leading to generation of second messengers | 0.101 | 32 | 9 | 8.78 | 8.8 |
| Chemokine receptors bind chemokines | 0.101 | 59 | 5 | 4.51 | 4.43 |
| Citric acid cycle (TCA cycle) | 0.1 | 22 | 4 | 10.5 | 10.5 |
| Other interleukin signaling | 0.0998 | 24 | 5 | 7.87 | 7.88 |
| RHO GTPases activate PAKs | 0.0927 | 24 | 4 | 11.5 | 11.5 |
| Ubiquitin-dependent degradation of Cyclin D (See also: Regulation of activated PAK-2p34 by proteasome mediated degradation) | 0.0918 | 53 | 6 | 10.8 | 10.8 |
| Interleukin-6 family signaling | 0.0914 | 24 | 3 | 7.69 | 7.66 |
| Keratan sulfate biosynthesis | 0.0913 | 28 | 5 | 7.64 | 7.65 |
| VEGFR2 mediated cell proliferation | 0.0902 | 21 | 4 | 10.2 | 10.2 |
| Degradation of AXIN | 0.0901 | 55 | 5 | 10.5 | 10.5 |
| mRNA Splicing - Minor Pathway | 0.0897 | 52 | 6 | 9.35 | 9.37 |
| Pre-NOTCH Processing in Golgi | 0.0886 | 18 | 3 | 9.86 | 9.87 |
| ROS and RNS production in phagocytes | 0.0875 | 36 | 6 | 7.95 | 8.08 |
| SUMOylation of DNA replication proteins | 0.0869 | 45 | 7 | 9.87 | 9.85 |
| SCF(Skp2)-mediated degradation of p27/p21 | 0.0869 | 60 | 5 | 10.8 | 10.8 |
| Degradation of DVL | 0.0865 | 57 | 5 | 10.5 | 10.5 |

**Supplementary Table 6.** Top 20 most significant pathways from differential network analysis using LUSC subsetting on samples with tumor purity above 70%. Columns include Reactome pathway names, differentially connectivity (DC) score, number of genes in the pathway, number of significant DC genes, and average expression level of genes in the pathway.

| Gene | logFC | Avg. expr. | BH adj.  p-value |
| --- | --- | --- | --- |
| DPY30 | 0.22 | 9.3 | 0.001 |
| MRPS23 | 0.22 | 9.7 | 0.003 |
| SEC11A | 0.18 | 10.9 | 0.003 |
| MRPL33 | 0.25 | 8.5 | 0.003 |
| POLR2G | 0.22 | 10.1 | 0.003 |

**Supplementary Table 7.** Five most significant DEGs from differential expression analysis using HNSC without subsetting.

| Gene | logFC | Avg. expr. | BH adj.  p-value |
| --- | --- | --- | --- |
| DPY30 | 0.3 | 9.4 | 0.010 |
| NSMCE2 | 0.33 | 8.6 | 0.035 |
| POLE4 | 0.39 | 9.5 | 0.035 |
| TNRC6B | -0.36 | 9.6 | 0.035 |
| POLR2G | 0.31 | 10.2 | 0.035 |

**Supplementary Table 8.** Five most significant DEGs from differential expression analysis using HNSC subset on samples with tumor purity above 70%.

| Gene | logFC | Avg. expr. | BH adj.  p-value |
| --- | --- | --- | --- |
| AMDHD2 | 0.31 | 7.7 | 0.02 |
| SIGIRR | 0.35 | 8.5 | 0.02 |
| UCKL1 | 0.21 | 9.5 | 0.025 |
| LCLAT1 | -0.22 | 9.5 | 0.057 |
| CEBPB | 0.32 | 10.2 | 0.057 |

**Supplementary Table 9.** Results from differential expression analysis using LUSC without subsetting.

| Gene | logFC | Avg. expr. | BH adj.  p-value |
| --- | --- | --- | --- |
| KCTD1 | -0.61 | 10.2 | 0.113 |
| UCKL1 | 0.29 | 9.6 | 0.271 |
| SIGIRR | 0.47 | 8.4 | 0.271 |
| IFI16 | -0.44 | 12.3 | 0.356 |
| CLDN3 | 1.12 | 6.4 | 0.356 |

**Supplementary Table 10.** Results from differential expression analysis using LUSC subset on samples with tumor purity above 70%.

| Pathway | DC score | # genes | # DC genes | Avg. expr.  in low-risk | Avg. expr.  in high-risk |
| --- | --- | --- | --- | --- | --- |
| MET activates PTK2 signaling | 0.0757 | 30 | 2 | -0.0311 | 0.0312 |
| Inflammasomes | 0.0732 | 23 | 4 | -0.00432 | 0.00434 |
| PD-1 signaling | 0.0717 | 23 | 3 | -0.00249 | 0.0025 |
| Listeria monocytogenes entry into host cells | 0.0715 | 21 | 1 | 0.0235 | -0.0237 |
| Regulation of ornithine decarboxylase (ODC) | 0.0713 | 51 | 7 | -0.00403 | 0.00405 |
| Eukaryotic Translation Elongation (See also: Peptide chain elongation) | 0.0713 | 93 | 13 | 0.0422 | -0.0424 |
| Degradation of AXIN | 0.071 | 55 | 6 | -0.00295 | 0.00297 |
| Ubiquitin-dependent degradation of Cyclin D (See also: Regulation of activated PAK-2p34 by proteasome mediated degradation) | 0.0708 | 53 | 9 | -0.00307 | 0.00309 |
| Regulation of Apoptosis (See also: Regulation of activated PAK-2p34 by proteasome mediated degradation) | 0.0707 | 53 | 7 | -0.00163 | 0.00164 |
| Negative regulation of NOTCH4 signaling | 0.0706 | 54 | 7 | -0.00396 | 0.00398 |
| Signal transduction by L1 | 0.0704 | 21 | 4 | -0.00231 | 0.00232 |
| Collagen chain trimerization | 0.0704 | 44 | 4 | -0.0198 | 0.02 |
| SCF(Skp2)-mediated degradation of p27/p21 | 0.0703 | 60 | 11 | -0.00531 | 0.00534 |
| RNA Polymerase III Transcription Initiation From Type 1 Promoter (See also: RNA Polymerase III Transcription Initiation From Type 2 Promoter) | 0.0703 | 28 | 5 | 0.00331 | -0.00333 |
| Complex I biogenesis | 0.0701 | 55 | 6 | -0.0078 | 0.00785 |
| NOTCH4 Intracellular Domain Regulates Transcription | 0.0701 | 20 | 1 | 0.0198 | -0.0199 |
| Vif-mediated degradation of APOBEC3G | 0.0699 | 55 | 6 | -0.00197 | 0.00198 |
| FBXL7 down-regulates AURKA during mitotic entry and in early mitosis | 0.0697 | 54 | 7 | -0.00424 | 0.00427 |
| Cross-presentation of soluble exogenous antigens (endosomes) | 0.0695 | 50 | 5 | -0.0126 | 0.0127 |
| Degradation of DVL | 0.0695 | 57 | 4 | -0.00537 | 0.0054 |

**Supplementary Table 11.** Top 20 most significant pathways from TP-adjusted differential network analysis on BRCA. Columns include Reactome pathway names, differentially connectivity (DC) score, number of genes in the pathway, number of significant DC genes, and average expression level of genes in the pathway.

| Pathway | DC score | # genes | # DC genes | Avg. expr.  in low-risk | Avg. expr.  in high-risk |
| --- | --- | --- | --- | --- | --- |
| Digestion | 0.0934 | 22 | 2 | 0.00642 | -0.0064 |
| Molecules associated with elastic fibres | 0.092 | 38 | 6 | -0.0618 | 0.0616 |
| Regulation of FZD by ubiquitination | 0.0895 | 21 | 2 | -0.0292 | 0.029 |
| mTORC1-mediated signalling | 0.0891 | 23 | 3 | 0.00308 | -0.00306 |
| Transport of the SLBP independent Mature mRNA (See also: Transport of the SLBP Dependant Mature mRNA) | 0.0886 | 35 | 7 | -0.0122 | 0.0122 |
| SHC1 events in ERBB2 signaling | 0.0886 | 22 | 5 | -0.000759 | 0.000756 |
| Digestion and absorption | 0.0885 | 27 | 1 | -0.00244 | 0.00244 |
| Nuclear Pore Complex (NPC) Disassembly | 0.0881 | 35 | 5 | -0.0101 | 0.01 |
| HDR through Single Strand Annealing (SSA) | 0.0875 | 37 | 2 | -0.0204 | 0.0204 |
| VEGFR2 mediated cell proliferation | 0.0872 | 21 | 2 | -0.00701 | 0.00698 |
| Apoptotic factor-mediated response | 0.0869 | 18 | 3 | 0.000389 | -0.000388 |
| Export of Viral Ribonucleoproteins from Nucleus (See also: NEP/NS2 Interacts with the Cellular Export Machinery) | 0.0866 | 39 | 5 | -0.0128 | 0.0127 |
| Other interleukin signaling | 0.0863 | 24 | 2 | -0.0117 | 0.0116 |
| RNA Polymerase III Transcription Termination | 0.0862 | 23 | 4 | 0.00792 | -0.00789 |
| RA biosynthesis pathway | 0.0851 | 22 | 4 | -0.04 | 0.0398 |
| Transport of Mature mRNA Derived from an Intronless Transcript (See also: Transport of Mature mRNAs Derived from Intronless Transcripts) | 0.0848 | 42 | 6 | -0.00678 | 0.00675 |
| Interactions of Vpr with host cellular proteins | 0.0847 | 40 | 4 | -0.00926 | 0.00923 |
| Host Interactions with Influenza Factors (See also: NS1 Mediated Effects on Host Pathways) | 0.0845 | 45 | 6 | -0.00465 | 0.00463 |
| Chondroitin sulfate/dermatan sulfate metabolism | 0.0837 | 50 | 2 | -0.0179 | 0.0178 |
| DAP12 interactions | 0.0825 | 43 | 4 | -0.0182 | 0.0181 |

**Supplementary Table 12.** Top 20 most significant pathways from TP-adjusted differential network analysis on HNSC. Columns include Reactome pathway names, differentially connectivity (DC) score, number of genes in the pathway, number of significant DC genes, and average expression level of genes in the pathway.

| Pathway | DC score | # genes | # DC genes | Avg. expr.  in low-risk | Avg. expr.  in high-risk |
| --- | --- | --- | --- | --- | --- |
| Resolution of D-loop Structures through Synthesis-Dependent Strand Annealing (SDSA) | 0.0928 | 26 | 5 | -0.032 | 0.032 |
| Syndecan interactions | 0.0928 | 27 | 3 | 0.02 | -0.02 |
| Generation of second messenger molecules | 0.0923 | 33 | 1 | 0.00808 | -0.00808 |
| ERCC6 (CSB) and EHMT2 (G9a) positively regulate rRNA expression | 0.0921 | 77 | 2 | -0.00219 | 0.00219 |
| Resolution of D-Loop Structures (See also: Resolution of D-loop Structures through Holliday Junction Intermediates) | 0.0887 | 34 | 2 | -0.0216 | 0.0216 |
| Meiotic recombination | 0.0874 | 86 | 3 | -0.00705 | 0.00705 |
| Assembly of collagen fibrils and other multimeric structures | 0.0871 | 61 | 4 | 0.0272 | -0.0272 |
| Pre-NOTCH Processing in Golgi | 0.0858 | 18 | 2 | 0.0166 | -0.0166 |
| Role of phospholipids in phagocytosis | 0.0843 | 25 | 4 | 0.0066 | -0.0066 |
| FGFR2 alternative splicing | 0.0842 | 26 | 3 | -0.00242 | 0.00242 |
| Cross-presentation of soluble exogenous antigens (endosomes) | 0.084 | 50 | 4 | 0.00117 | -0.00117 |
| Ubiquitin-dependent degradation of Cyclin D (See also: Regulation of activated PAK-2p34 by proteasome mediated degradation) | 0.084 | 53 | 2 | -0.00195 | 0.00195 |
| Platelet calcium homeostasis | 0.0834 | 30 | 6 | 0.0202 | -0.0202 |
| Regulation of Apoptosis (See also: Regulation of activated PAK-2p34 by proteasome mediated degradation) | 0.0831 | 53 | 3 | -0.00325 | 0.00325 |
| Interleukin-10 signaling | 0.0829 | 47 | 7 | 0.0303 | -0.0303 |
| CDT1 association with the CDC6:ORC:origin complex | 0.0823 | 59 | 4 | -0.00345 | 0.00345 |
| Synthesis of PC | 0.082 | 28 | 6 | 0.008 | -0.008 |
| Defective CFTR causes cystic fibrosis | 0.0819 | 61 | 8 | -0.00183 | 0.00183 |
| Degradation of DVL | 0.0816 | 57 | 5 | 0.00212 | -0.00212 |
| Metabolism of polyamines | 0.081 | 59 | 4 | -0.00162 | 0.00162 |

**Supplementary Table 13.** Top 20 most significant pathways from TP-adjusted differential network analysis on LUSC. Columns include Reactome pathway names, differentially connectivity (DC) score, number of genes in the pathway, number of significant DC genes, and average expression level of genes in the pathway.

| Gene | logFC | Avg. expr. | BH adj.  p-value |
| --- | --- | --- | --- |
| DPY30 | 0.21 | 9.3 | 0.001 |
| PPIB | 0.26 | 13.2 | 0.003 |
| MRPS23 | 0.21 | 9.7 | 0.003 |
| FGFR2 | -0.50 | 9.6 | 0.003 |
| SEC11A | 0.18 | 10.9 | 0.003 |

**Supplementary Table 14.** Five most significant DEGs from TP-adjusted differential expression analysis using HNSC.

| Gene | logFC | Avg. expr. | BH adj.  p-value |
| --- | --- | --- | --- |
| UCKL1 | 0.22 | 9.5 | 0.015 |
| AMDHD2 | 0.3 | 7.7 | 0.015 |
| SIGIRR | 0.34 | 8.5 | 0.015 |
| LCLAT1 | -0.21 | 9.5 | 0.026 |
| CYBA | 0.34 | 10.8 | 0.026 |

**Supplementary Table 15.** Five most significant DEGs from TP-adjusted differential expression analysis using LUSC.
